# Supplementary material for: The 6-Month Antibody Durability of Heterologous Convidecia Plus CoronaVac and Homologous CoronaVac Immunizations in People Aged 18–59 Years and over 60 Years Based on Two Randomized Controlled Trials in China
Source: Vaccines (Basel). 2023 Dec 4;11(12):1815. doi: 10.3390/vaccines11121815 (PMC10747853; doi:10.3390/vaccines11121815)
Supplement: Supplementary file 1 [file vaccines-11-01815-s001.zip › vaccines-2664862-supplementary.pdf]

Supplementary Materials

Table S1. The GMT, and seropositivity of neutralizing antibodies to SARS-CoV-2 prototype and RBD-specific IgG antibodies at day 14 and at month 6 of people aged 18-59 years and over 60 years.

	**Group A**	**Group B**	***P* value**	**Group C**	**Group D**	***P* value**
Aged 18-59 years						
Wild-type						
Day 14	n=96	n=102		n=51	n=49	
GMT	197.4(167.6-232.4)	33.6(28.3-40.0)	<0.001	53.4(37.9-78.0)	12.8(9.3-17.5)	<0.001
Seropositivity	100.0(96.2-100.0)	100.0(96.4-100.0)	-	98.0(89.6-100)	87.8(75.2-95.4)	0.057
Month 6	n=90	n=95		n=47	n=44	
GMT	30.6(25.1-37.2)	6.9(5.6-8.6)	<0.001	8.5(6.2-11.7)	2.7(2.3-3.1)	<0.001
Seropositivity	96.7(90.6-99.3)	72.6(62.5-81.3)	<0.001	78.7(64.3-89.3)	27.1(15.3-41.8)	<0.001
Anti-RBD IgG						
Day 14	n=96	n=102		n=51	n=49	
GMT	3090.30(2636.3-2622.4)	369.0(304.2-447.5)	<0.001	941.9(663.9-1336.0)	154.1(116.3-204.3)	<0.001
Seropositivity	100.0(96.2-100)	100.0(96.0-100.0)	-	100.0(93.0-100.0)	100.0(92.7-100.0)	-
Month 6	n=90	n=95		n=47	n=44	
GMT	270.0(226.3-322.1)	60.5(48.6-75.4)	<0.001	41.7(29.9-58.1)	14.6(11.5-18.5)	<0.001
Seropositivity	100.0(96.0-100.0)	100.0(96.2-100.0)	-	93.6(82.5-98.7)	85.4(72.2-93.9)	0.196
Aged over 60 years						
Wild-type						
Day 14	n=102	n=100		n=48	n=47	
GMT	301.2(266.4-340.6)	48.2(39.5-58.7)	<0.001	71.0(49.5-101.7)	9.3(6.2-13.9)	<0.001
Seropositivity	100.0(96.3-100.0)	99.0(94.6-100)	0.321	97.9(88.9-100)	68.1(52.9-80.9)	<0.001
Month 6	n=84	n=86		n=45	n=44	
GMT	49.1(38.0-63.6)	9.4(7.7-11.4)	<0.001	11.6(8.4-16.0)	3.3(2.7-4.0)	<0.001
Seropositivity	100.0(95.7-100.0)	93.0(85.4-97.4)	0.014	91.1(78.8-97.5)	40.9(26.3-56.8)	<0.001
Anti-RBD						
Day 14	n=102	n=100		n=48	n=47	
GMT	3180.5(2609.8-3875.2)	428.4(355.5-516.1)	<0.001	1280.3(794.3-1828.5)	146.5(88.3-243.1)	<0.001
Seropositivity	100.0(96.3-100.0)	100.0(96.4-100.0)	-	100.0(92.6-100.0)	89.4(76.9-96.5)	0.02
Month 6	n=84	n=86		n=45	n=44	
GMT	311.8(240.8,403.7)	47.1(37.4,59.3)	0.005	52.3(37.9,72.1)	17.3(12.7-23.8)	0.0016
Seropositivity	100.0(95.7-100.0)	98.8(93.7-100.0)	0.322	95.6(84.9-99.5)	75.0(59.7-86.8)	<0.001

Data are GMT (95% CI), seropositivity (%, 95% CI). Group A=Participants completed the two-dose primary series of CoronaVac and a booster dose of Convidecia, Group B= Participants completed the two-dose primary series of CoronaVac and a booster dose of CoronaVac, Group C=Participants received one-dose of CoronaVac and another dose of Convidecia, Group D=Participants

received one-dose of CoronaVac and another dose of CoronaVac. GMT=geometric mean titer.

Table S2. The GMT and seropositivity of neutralizing antibodies to the Delta and Omicron(B.1.1.529) variants at day 14 and at month 6 of people aged 18-59 years and over 60 years.

	Group A	Group B	*P* value	Group C	Group D	*P* value
Aged 18-59 years						
Delta						
Day 14	n=96	n=102		n=51	n=50	
GMT	55.0(44.5-68.0)	8.2(6.6-10.1)	<0.001	10.8(7.1-16.5)	3.6(2.9-4.4)	0.039
Seropositivity	99.0(88.4-100.0)	78.4(54.1-87.7)	<0.001	70.6(32.3-83.7)	44.9(16.3-67.7)	0.009
Month 6	n=90	n=95		n=47	n=44	
GMT	11.7(9.6-14.1)	5.2(4.5-5.9)	<0.001	4.8(3.8-6.1)	3.0(2.6-3.3)	<0.001
Seropositivity	96.7(90.6-99.3)	85.3(76.5-91.7)	0.007	76.6(62.0-87.7)	52.1(37.2-66.7)	0.013
Rate difference	2.3(-1.9-6.5)	-6.9(-17.6-3.8)		-6.0(-23.4-11.4)	-7.2(-27.4-13.0)	
Omicron(B.1.1.529)						
Day 14	n=30	n=30		n=15	n=15	
GMT	21.6(14.3-32.6)	4.7(3.0-7.2)	<0.001	2.0(2.0-2.0)	2.0(2.0-2.0)	-
Seropositivity	90.0(73.5-97.9)	53.3(34.3-71.7)	0.002	0	0	-
Month 6	n=90	n=95		n=47	n=44	
GMT	2.0(2.0-2.0)	2.0(2.0-2.0)	0.558	2.0(2.0-2.0)	2.0(2.0-2.0)	-
Seropositivity	2.2(0.3-7.8)	2.1(0.3-7.4)	0.956	0	0	-
Rate difference	87.8(76.7-99.0)	51.2(33.1-69.3)		0	0	
Aged over 60years						
Delta						
Day 14	n=44	n=40		n=23	n=21	
GMT	43.2(31.5-59.2)	7.9(5.8-10.7)	<0.001	5.1(3.2-8.2)	4.3(2.45-7.4)	0.619
Seropositivity	100.0(92.0-100.0)	80.0(64.4-90.9)	0.002	56.5(34.5-76.8)	33.3(14.6-57.0)	0.123
Month 6	n=84	n=86		n=45	n=44	
GMT	9.2(6.9-12.3)	2.7(2.4-3.0)	<0.001	2.2(2.0-2.5)	2.1(2.0-2.2)	0.196
Seropositivity	64.3(53.1-74.4)	26.6(17.8-37.4)	<0.001	11.1(3.7-24.1)	4.5(0.6-15.5)	0.250
Rate difference	35.7(25.5-46.0)	53.4(37.9-68.9)		45.4(23.2-67.6)	28.8(7.7-49.9)	
Omicron(B.1.1.529)						
Day 14	n=44	n=40		n=23	n=21	
GMT	28.2(19.3-41.3)	4.1(3.0-5.7)	<0.001	4.9(2.8-8.7)	3.9(2.2-6.8)	0.529
Seropositivity	93.2(81.3-98.6)	40.0(24.9-56.7)	<0.001	47.8(26.8-69.4)	33.3(14.6-57.0)	0.329
Month 6	n=84	n=86		n=45	n=44	
GMT	3.0(2.4-3.6)	2.2(2.0-2.4)	0.005	2.0(2.0-2.0)	2.0(2.0-2.0)	-
Seropositivity	17.9(10.4-27.7)	8.1(3.3-16.1)	0.059	0	0	-
Rate difference	75.3(64.2-86.4)	31.9(15.7-48.1)		47.8(27.4-68.2)	33.0(12.9-53.1)	

Data are GMT (95% CI), seropositivity(%, 95% CI), rate difference(%, 95%CI, two time points of day 14 and month 6). Group A=Participants completed the two-dose primary series of CoronaVac and a booster dose of Convidecia, Group B= Participants completed the two-dose primary series of CoronaVac and a booster dose of CoronaVac, Group C=Participants received one-dose of CoronaVac and another dose of Convidecia, Group D=Participants received one-dose of CoronaVac and another dose of CoronaVac. GMT=geometric mean titer.

Table S3. The GMT and seropositivity of pseudovirus neutralizing antibodies to the Omicron(BA.4/5) variant at day 14 and at month 6 of people aged 18-59 years and over 60 years.

	Group A	Group B	*P* value	Group C	Group D	*P* value
Aged over 18-59 years						
Day 14	n=30	n=30		n=15	n=15	
GMT	37.8(30.4-46.9)	19.8(16.4-24.0)	<0.001	22.8(16.9-30.8)	17.8(14.6-21.6)	0.109
Seropositivity	80.0(61.4-92.3)	26.7(12.3-45.9)	<0.001	40.0(16.3-67.7)	20.0(4.3-48.1)	0.157
Month 6	n=30	n=30		n=15	n=15	
GMT	19.6(16.7-23.0)	18.9(16.3-22.0)	0.856	17.8(14.6-21.6)	19.7(13.6-28.6)	0.724

Seropositivity	26.7(12.3-45.9)	26.7(12.3-45.9)	0.937	20.0(4.3-48.1)	20.0(4.3-48.1)	0.739
Rate difference	53.3(32.0-74.6)	0(-22.4-22.4)		20.0(-12.0-52.1)	0(-28.6-28.6)	
Aged over 60 years						
Day 14	n=49	n=50		n=25	n=23	
GMT	65.5(47.8-90.1)	44.0(30.1-64.4)	0.109	42.0(29.2-60.4)	37.8(23.5-60.9)	0.367
Seropositivity	75.5(61.1-86.7)	58.0(43.2-71.8)	0.065	68.0(46.5-85.1)	60.9(38.5-80.3)	0.606
Month 6	n=44	n=40		n=24	n=22	
GMT	33.5(25.1-44.8)	44.4(32.4-60.9)	0.190	25.9(18.7-35.8)	24.4(19.1-31.2)	0.292
Seropositivity	54.4(38.8-69.6)	65.0(48.3-79.4)	0.330	41.7(22.1-63.4)	45.5(24.4-67.8)	0.796
Rate difference	21.1(2.1-40.1)	-7.0(-27.1-13.1)		26.3(-0.6-53.2)	15.4(-13.4-44.2)	

Data are GMT (95% CI), seropositivity(%, 95% CI), rate difference(%, 95%CI, two time points of day 14 and month 6). Group A=Participants completed the two-dose primary series of CoronaVac and a booster dose of Convidecia, Group B= Participants completed the two-dose primary series of CoronaVac and a booster dose of CoronaVac, Group C=Participants received one-dose of CoronaVac and another dose of Convidecia, Group D=Participants received one-dose of CoronaVac and another dose of CoronaVac. GMT=geometric mean titer.

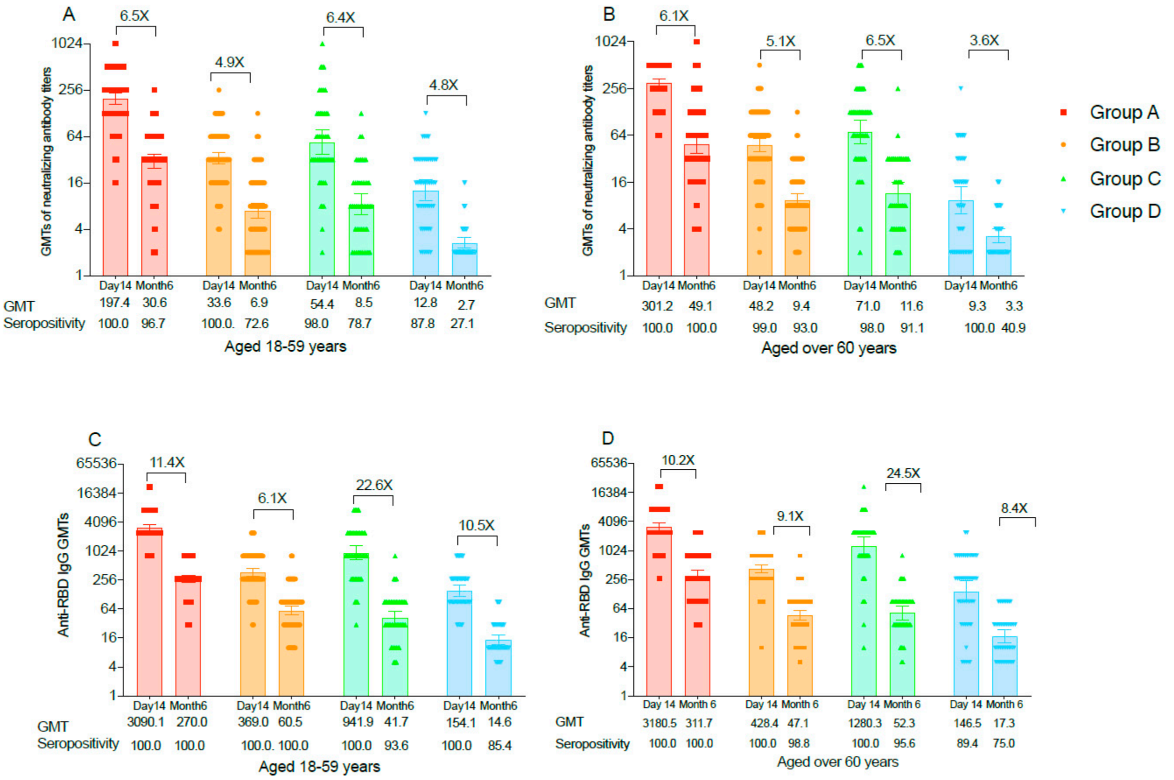

Figure S1. The neutralising antibodies against SARS-CoV-2 prototype and RBD-specific IgG antibodies at day 14 and at month 6 of people aged 18-59 years and over 60 years . Group A=Participants completed the two-dose primary series of CoronaVac and a booster dose of Convidecia, Group B= Participants completed the two-dose primary series of CoronaVac and a booster dose of CoronaVac, Group C=Participants received one-dose of CoronaVac and another dose of Convidecia, Group D=Participants received one-dose of CoronaVac and another dose of CoronaVac. Data above the bars show the reduction folds of GMTs of antibodies titers at month 6 compared to day 14. GMT=geometric mean titer.

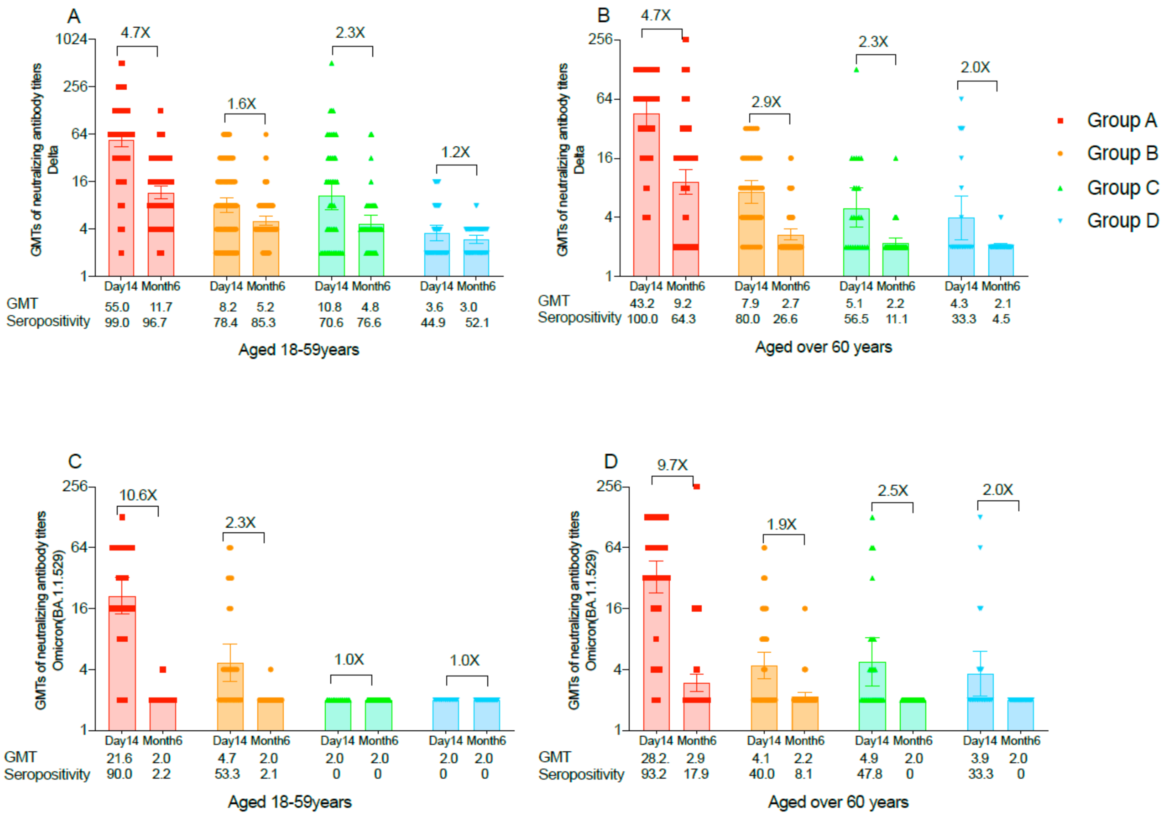

Figure S2. The neutralising antibodies against the Delta and Omicron(B.1.1.529) variants at day 14 and at month 6 of people aged 18-59 years and over 60 years . Group A=Participants completed the two-dose primary series of CoronaVac and a booster dose of Convidecia, Group B= Participants completed the two-dose primary series of CoronaVac and a booster dose of CoronaVac, Group C=Participants received one-dose of CoronaVac and another dose of Convidecia, Group D=Participants received one-dose of CoronaVac and another dose of CoronaVac. Data above the bars show the reduction folds of GMTs of antibodies titers at month 6 compared to day 14. GMT=geometric mean titer.

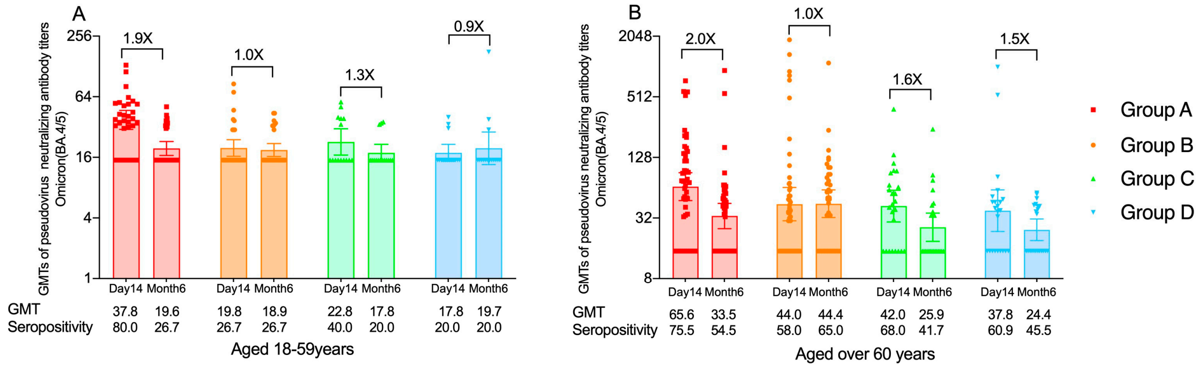

Figure S3. The pseudovirus neutralising antibodies against the Omicron(BA.4/5)variant at day 14 and at month 6 of people aged 18-59 years and over 60 years . Group A=Participants completed the two-dose primary series of CoronaVac and a booster dose of Convidecia, Group B= Participants completed the two-dose primary series of CoronaVac and a booster dose of CoronaVac, Group C=Participants received one-dose of CoronaVac and another dose of Convidecia, Group D=Participants received one-dose of CoronaVac and another dose of CoronaVac. Data above the bars show the reduction folds of

GMTs of antibodies titers at month 6 compared to day 14. GMT=geometric mean titer.

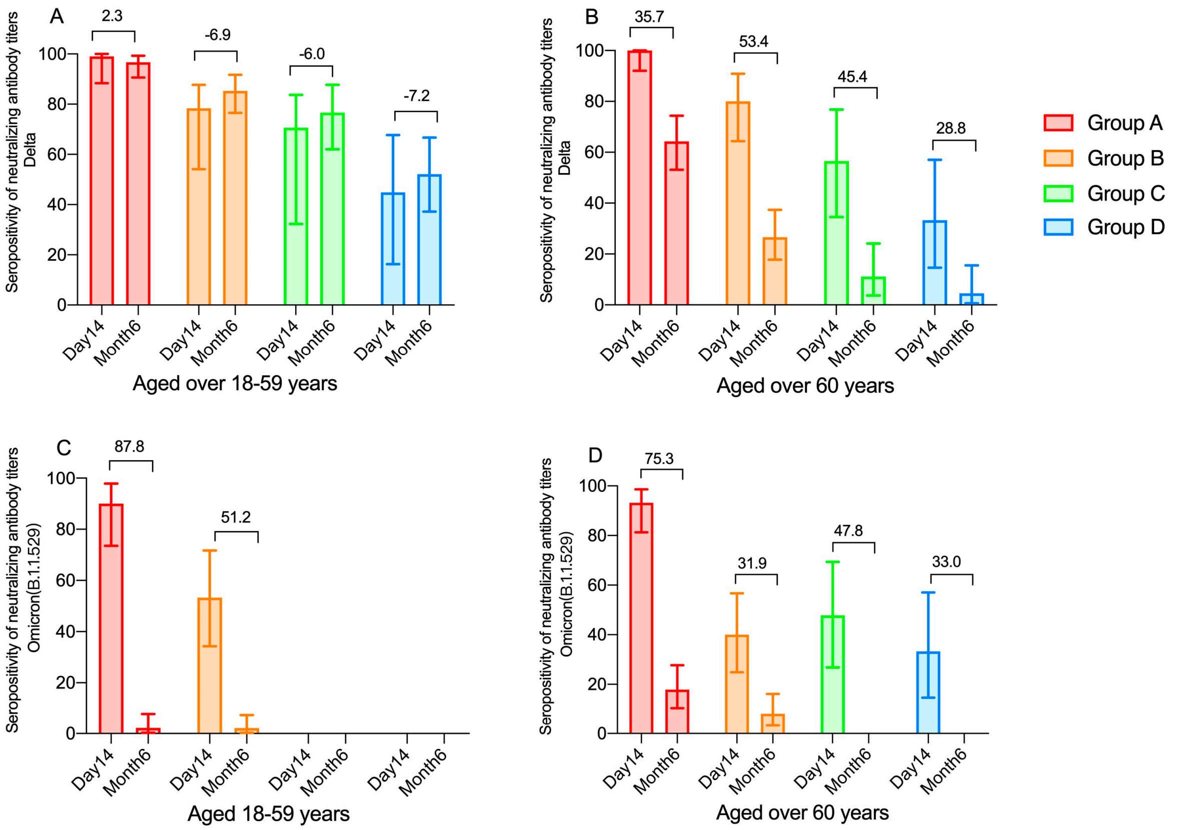

Figure S4. The seropositivity of neutralising antibodies against the Delta and Omicron(B.1.1.529) variants at day 14 and at month 6 of people aged 18-59 years and over 60 years . Group A=Participants completed the two-dose primary series of CoronaVac and a booster dose of Convidecia, Group B= Participants completed the two-dose primary series of CoronaVac and a booster dose of CoronaVac, Group C=Participants received one-dose of CoronaVac and another dose of Convidecia, Group D=Participants received one-dose of CoronaVac and another dose of CoronaVac. Data above the bars show the seropositive rate difference of day 14 and month 6.

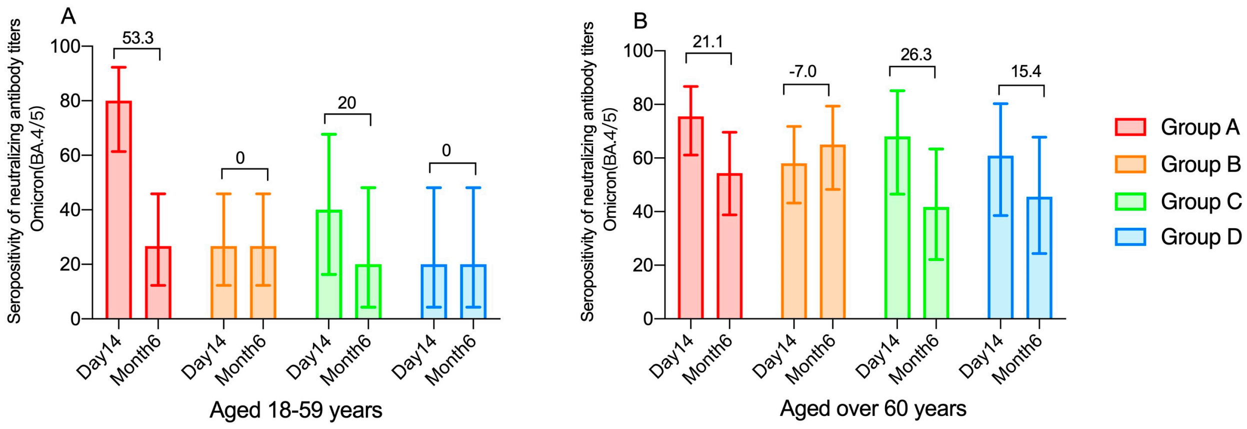

Figure S5. The seropositivity of pseudovirus neutralising antibodies against the Omicron(BA.4/5) variants at day 14 and at month 6 of people aged 18-59 years and over 60 years . Group A=Participants completed the two-dose primary series of CoronaVac and a booster dose of Convidecia, Group B= Participants

completed the two-dose primary series of CoronaVac and a booster dose of CoronaVac, Group C=Participants received one-dose of CoronaVac and another dose of Convidecia, Group D=Participants received one-dose of CoronaVac and another dose of CoronaVac. Data above the bars show the seropositive rate difference of day 14 and month 6.

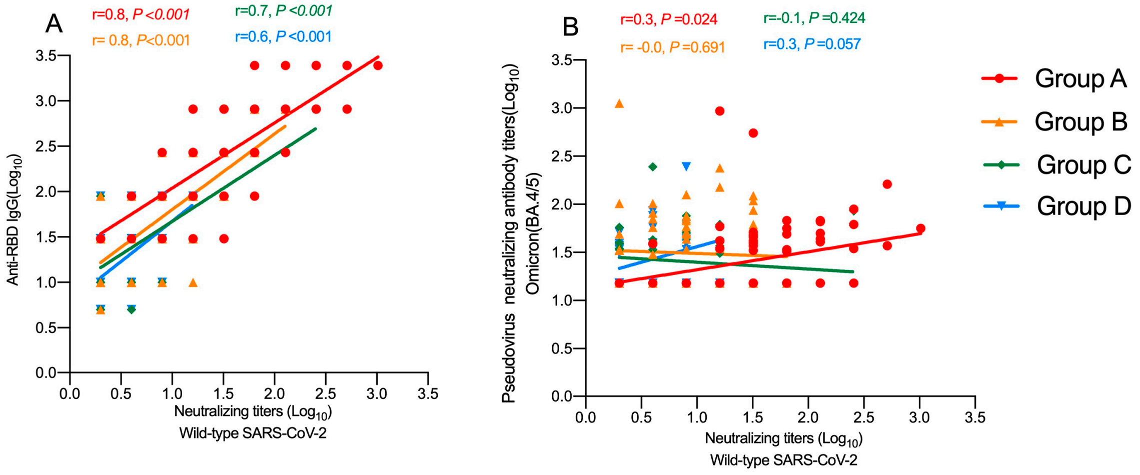

Figure S6. The correlation analyzed between the neutralising antibodies against SARS-CoV-2 prototype and RBD-specific IgG antibodies and the pseudovirus neutralising antibodies against the Omicron(BA.4/5)variant at month 6 of people aged 18-59 years and over 60 years. Group A=Participants completed the two-dose primary series of CoronaVac and a booster dose of Convidecia, Group B= Participants completed the two-dose primary series of CoronaVac and a booster dose of CoronaVac, Group C=Participants received one-dose of CoronaVac and another dose of Convidecia, Group D=Participants received one-dose of CoronaVac and another dose of CoronaVac. Pearson correlation coefficients are presented for each regimen.
